# Supplementary material for: Relationship Between Dietary Habits and Stress Responses Exerted by Different Gut Microbiota
Source: Nutrients. 2025 Apr 20;17(8):1388. doi: 10.3390/nu17081388 (PMC12030070; doi:10.3390/nu17081388)
Supplement: Supplementary file 1 [file nutrients-17-01388-s001.zip › nutrients-3574068-supplementary.pdf]

Supplementary materials

Table S1. Evaluation of the number of clusters

# of clusters	Silhouette coefficient	Calinski-Harabasz index	Davies-Bouldin index
2	0.104	35.5	2.51
3	0.092	56.2	2.17
4	<u>0.142</u>	<u>123.5</u>	1.94
5	0.132	96.0	<u>1.74</u>
6	0.061	88.8	2.16
7	0.092	98.8	2.09
8	0.091	86.5	1.97
9	0.062	78.7	2.13

Note: The measure of similarity is Jensen-Shannon divergence, and the linkage method is complete linkage. Underlined the local extreme values for each indicator.

Figure S1. Cluster analysis of genus-level gut microbiota composition ratios

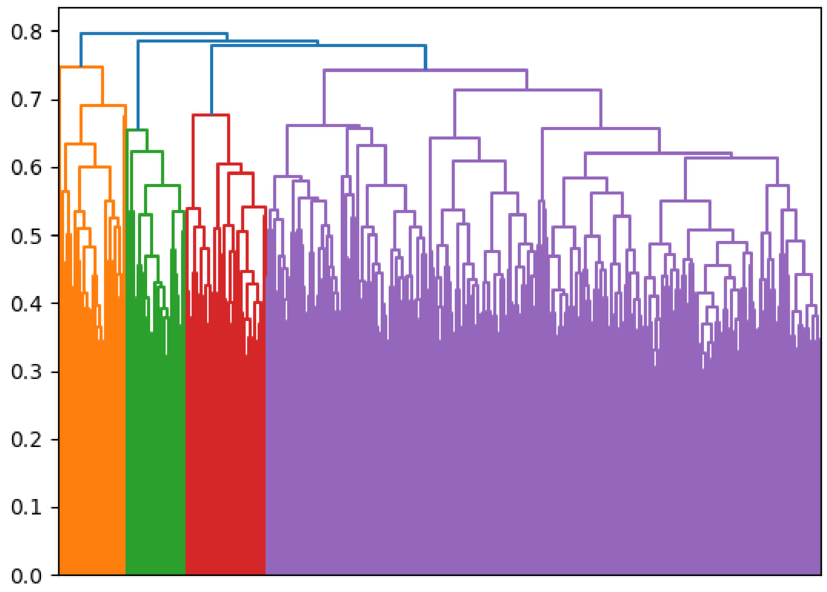

Note: The number of participants is n=821.

Table S2. Estimation results of regression analysis

	Vigor				Irritability				Fatigue			
N	787				787				787			
R2	0.316				0.364				0.489			
adj. R2	0.259				0.311				0.447			
F	5.59				6.92				11.60			
p>F	0.000				0.000				0.000			
	Coef.	[95%	C.I.]	p-value	Coef.	[95%	C.I.]	p-value	Coef.	[95%	C.I.]	p-value
Cluster1 × cereals	0.062	-0.606	0.731	0.855	0.325	-0.240	0.891	0.259	0.451	-0.097	0.999	0.107
Cluster2 × cereals	0.262	-0.488	1.012	0.493	-0.539	-1.174	0.095	0.096 *	0.260	-0.356	0.876	0.407
Cluster3 × cereals	0.086	-0.367	0.540	0.708	-0.101	-0.485	0.283	0.606	0.359	-0.012	0.730	0.058 *
Cluster4 × cereals	0.081	-0.136	0.299	0.462	-0.005	-0.190	0.179	0.954	0.109	-0.070	0.287	0.232
Cluster1 × potatoes	-0.844	-1.667	-0.020	0.045 **	-0.414	-1.114	0.285	0.245	-0.328	-1.006	0.350	0.343
Cluster2 × potatoes	-0.148	-1.197	0.901	0.781	-0.292	-1.181	0.597	0.520	-0.715	-1.575	0.145	0.103
Cluster3 × potatoes	-0.195	-0.779	0.388	0.511	0.162	-0.332	0.657	0.519	-0.115	-0.594	0.364	0.637
Cluster4 × potatoes	0.138	-0.085	0.362	0.225	-0.056	-0.246	0.134	0.565	0.125	-0.058	0.309	0.181
Cluster1 × beans	0.291	-0.590	1.172	0.516	0.371	-0.375	1.117	0.330	0.385	-0.338	1.109	0.296
Cluster2 × beans	0.227	-0.519	0.973	0.550	0.316	-0.316	0.948	0.326	0.099	-0.514	0.712	0.751
Cluster3 × beans	-0.003	-0.565	0.558	0.991	0.299	-0.177	0.774	0.218	0.483	0.024	0.943	0.039 **
Cluster4 × beans	0.184	0.005	0.363	0.044 **	0.057	-0.095	0.209	0.460	-0.072	-0.220	0.075	0.336
Cluster1 × green yellow vegetables	0.530	-0.176	1.236	0.141	0.427	-0.172	1.025	0.162	0.156	-0.425	0.737	0.598
Cluster2 × green yellow vegetables	-0.362	-1.018	0.294	0.279	0.148	-0.408	0.704	0.602	-0.187	-0.726	0.352	0.496
Cluster3 × green yellow vegetables	0.255	-0.093	0.602	0.150	0.063	-0.232	0.357	0.677	0.000	-0.286	0.285	0.998
Cluster4 × green yellow vegetables	-0.218	-0.423	-0.014	0.036 **	-0.059	-0.233	0.115	0.506	-0.049	-0.217	0.120	0.571
Cluster1 × other vegetables	-0.064	-1.149	1.021	0.908	0.352	-0.567	1.272	0.452	0.116	-0.775	1.008	0.798
Cluster2 × other vegetables	0.189	-0.923	1.300	0.739	-0.578	-1.519	0.363	0.228	1.597	0.692	2.503	0.001 ***
Cluster3 × other vegetables	0.100	-0.331	0.531	0.649	-0.245	-0.610	0.120	0.188	0.006	-0.348	0.360	0.973
Cluster4 × other vegetables	0.088	-0.113	0.289	0.390	-0.009	-0.179	0.162	0.919	0.049	-0.117	0.214	0.564
Cluster1 × fruits	-0.462	-1.140	0.216	0.181	-0.593	-1.167	-0.019	0.043 **	-0.042	-0.600	0.516	0.883
Cluster2 × fruits	0.730	-0.246	1.707	0.142	-0.907	-1.733	-0.081	0.032 **	0.190	-0.614	0.993	0.643
Cluster3 × fruits	-0.217	-0.899	0.466	0.533	-0.144	-0.722	0.435	0.626	0.312	-0.248	0.873	0.274
Cluster4 × fruits	0.205	-0.067	0.477	0.140	-0.174	-0.405	0.057	0.139	-0.081	-0.305	0.143	0.477
Cluster1 × mushrooms	0.082	-0.714	0.877	0.841	-0.014	-0.688	0.661	0.968	0.292	-0.362	0.945	0.381
Cluster2 × mushrooms	-0.243	-0.928	0.441	0.486	0.765	0.187	1.342	0.010 ***	-0.318	-0.881	0.244	0.267
Cluster3 × mushrooms	-0.464	-1.325	0.397	0.291	-0.004	-0.735	0.726	0.991	-0.520	-1.227	0.187	0.149
Cluster4 × mushrooms	-0.166	-0.363	0.030	0.097 *	-0.096	-0.263	0.071	0.259	-0.142	-0.304	0.020	0.085 *
Cluster1 × seaweeds	-1.294	-2.316	-0.273	0.013 **	0.020	-0.849	0.890	0.963	-0.240	-1.083	0.603	0.576
Cluster2 × seaweeds	-0.031	-1.544	1.482	0.968	-1.358	-2.637	-0.080	0.037 **	-0.423	-1.665	0.820	0.504
Cluster3 × seaweeds	-0.092	-0.560	0.375	0.699	-0.324	-0.720	0.072	0.108	-0.081	-0.466	0.303	0.678
Cluster4 × seaweeds	0.025	-0.181	0.231	0.812	0.187	0.013	0.361	0.036 **	0.046	-0.124	0.215	0.596
Cluster1 × seafood	0.223	-0.439	0.885	0.509	-0.135	-0.696	0.426	0.636	0.190	-0.353	0.734	0.492
Cluster2 × seafood	0.222	-0.486	0.930	0.538	-0.554	-1.153	0.045	0.070 *	-0.194	-0.775	0.388	0.514
Cluster3 × seafood	0.477	-0.050	1.004	0.076 *	-0.321	-0.768	0.126	0.160	0.187	-0.247	0.621	0.397
Cluster4 × seafood	0.125	-0.078	0.328	0.227	-0.109	-0.282	0.063	0.212	-0.035	-0.202	0.132	0.683
Cluster1 × meat	1.161	0.481	1.841	0.001 ***	-0.364	-0.945	0.216	0.218	-0.737	-1.298	-0.177	0.010 ***
Cluster2 × meat	0.163	-0.208	0.534	0.388	0.196	-0.119	0.510	0.222	0.439	0.136	0.742	0.005 ***
Cluster3 × meat	0.159	-0.371	0.689	0.555	-0.040	-0.489	0.409	0.861	0.512	0.078	0.946	0.021 **
Cluster4 × meat	0.178	-0.021	0.378	0.080 *	-0.190	-0.358	-0.021	0.028 **	0.155	-0.008	0.319	0.063 *
Cluster1 × eggs	0.436	-0.278	1.150	0.231	-0.397	-1.002	0.208	0.198	-0.671	-1.257	-0.086	0.025 **
Cluster2 × eggs	0.417	-0.070	0.904	0.093 *	-0.201	-0.614	0.213	0.341	-0.020	-0.421	0.381	0.921
Cluster3 × eggs	0.202	-0.279	0.684	0.410	0.096	-0.312	0.505	0.643	0.161	-0.234	0.557	0.424
Cluster4 × eggs	0.013	-0.172	0.198	0.889	-0.142	-0.298	0.015	0.076 *	-0.093	-0.245	0.059	0.229
Cluster1 × dairy products	0.192	-0.460	0.844	0.564	-0.174	-0.726	0.379	0.537	-0.178	-0.714	0.357	0.514
Cluster2 × dairy products	0.662	-0.369	1.693	0.208	0.084	-0.791	0.959	0.850	-0.259	-1.107	0.589	0.549
Cluster3 × dairy products	0.270	-0.219	0.759	0.279	-0.281	-0.695	0.133	0.183	0.175	-0.226	0.577	0.391
Cluster4 × dairy products	0.114	-0.090	0.318	0.273	0.200	0.028	0.373	0.023 **	0.038	-0.129	0.206	0.653
Cluster1 × alcohol	-0.099	-0.601	0.404	0.700	-0.103	-0.529	0.323	0.635	0.093	-0.319	0.506	0.657
Cluster2 × alcohol	0.115	-0.652	0.883	0.768	0.313	-0.337	0.963	0.345	0.079	-0.552	0.709	0.807
Cluster3 × alcohol	-0.033	-0.644	0.577	0.914	-0.048	-0.566	0.470	0.855	0.149	-0.353	0.651	0.560
Cluster4 × alcohol	0.282	0.087	0.477	0.005 ***	0.145	-0.021	0.310	0.088 *	-0.045	-0.206	0.116	0.584
Female	-0.078	-0.737	0.580	0.815	0.845	0.290	1.400	0.003 ***	0.342	-0.199	0.883	0.215
Age	-0.002	-0.017	0.012	0.746	-0.023	-0.035	-0.010	0.000 ***	-0.029	-0.040	-0.017	0.000 ***
Living alone	0.249	-0.171	0.669	0.245	-0.718	-1.070	-0.365	0.000 ***	-0.137	-0.483	0.208	0.436
Vigor					0.005	-0.057	0.067	0.879	-0.063	-0.123	-0.004	0.037 **
Irritability	0.007	-0.079	0.093	0.879					0.104	0.034	0.174	0.004 ***
Fatigue	-0.094	-0.182	-0.006	0.037 **	0.111	0.036	0.186	0.004 ***				
Anxiety	0.055	-0.049	0.160	0.298	0.276	0.190	0.363	0.000 ***	0.238	0.154	0.322	0.000 ***
Depression	-0.325	-0.391	-0.260	0.000 ***	0.105	0.046	0.163	0.000 ***	0.142	0.086	0.199	0.000 ***
Physical	-0.038	-0.080	0.003	0.072 *	0.005	-0.030	0.041	0.763	0.112	0.079	0.145	0.000 ***
Const.	11.247	10.019	12.476	0.000 ***	3.392	2.164	4.619	0.000 ***	2.326	1.125	3.528	0.000 ***

Table S2. Estimation results of regression analysis (Cont'd.)

	Anxiety					Depression					Physical				
N	787					787					787				
R2	0.536					0.612					0.384				
adj. R2	0.498					0.580					0.334				
F	13.99					19.10					7.56				
p>F	0.000					0.000					0.000				
	Coef.	[95%	C.I.]	p-value		Coef.	[95%	C.I.]	p-value		Coef.	[95%	C.I.]	p-value	
Cluster1 × cereals	0.147	-0.318	0.612	0.535		-0.547	-1.244	0.151	0.124		-0.706	-1.876	0.463	0.236	
Cluster2 × cereals	0.017	-0.506	0.539	0.950		0.346	-0.438	1.130	0.387		0.754	-0.558	2.067	0.260	
Cluster3 × cereals	0.062	-0.253	0.378	0.698		-0.308	-0.781	0.166	0.202		-0.429	-1.222	0.365	0.289	
Cluster4 × cereals	0.190	0.039	0.341	0.014	**	-0.134	-0.361	0.093	0.247		0.059	-0.322	0.440	0.759	
Cluster1 × potatoes	0.195	-0.380	0.771	0.505		-0.281	-1.145	0.582	0.523		-0.329	-1.775	1.118	0.656	
Cluster2 × potatoes	0.352	-0.378	1.083	0.344		-0.400	-1.497	0.696	0.474		0.426	-1.412	2.263	0.649	
Cluster3 × potatoes	-0.280	-0.686	0.126	0.176		0.119	-0.491	0.729	0.703		1.054	0.035	2.073	0.043	**
Cluster4 × potatoes	0.005	-0.151	0.161	0.946		-0.026	-0.260	0.208	0.827		0.034	-0.358	0.426	0.865	
Cluster1 × beans	0.098	-0.516	0.712	0.755		0.207	-0.714	1.129	0.659		-2.490	-4.022	-0.957	0.001	***
Cluster2 × beans	0.042	-0.478	0.561	0.875		-0.181	-0.961	0.599	0.649		-0.077	-1.385	1.230	0.907	
Cluster3 × beans	-0.322	-0.712	0.069	0.106		-0.373	-0.960	0.213	0.212		0.603	-0.380	1.585	0.229	
Cluster4 × beans	0.011	-0.115	0.136	0.869		0.213	0.026	0.400	0.026	**	0.115	-0.199	0.430	0.473	
Cluster1 × green yellow vegetables	-0.049	-0.541	0.444	0.846		-0.151	-0.891	0.589	0.689		-0.893	-2.130	0.344	0.157	
Cluster2 × green yellow vegetables	0.154	-0.304	0.611	0.509		-0.049	-0.735	0.638	0.890		0.000	-1.150	1.151	0.999	
Cluster3 × green yellow vegetables	-0.052	-0.294	0.190	0.674		-0.027	-0.390	0.337	0.886		0.257	-0.351	0.866	0.407	
Cluster4 × green yellow vegetables	0.115	-0.027	0.258	0.112		-0.309	-0.522	-0.096	0.005	***	-0.041	-0.400	0.318	0.824	
Cluster1 × other vegetables	0.193	-0.563	0.949	0.616		-0.718	-1.852	0.415	0.214		2.160	0.265	4.054	0.026	**
Cluster2 × other vegetables	-0.308	-1.082	0.466	0.435		0.054	-1.109	1.217	0.927		-1.811	-3.754	0.132	0.068	*
Cluster3 × other vegetables	0.339	0.040	0.639	0.026	**	0.004	-0.448	0.455	0.988		-0.391	-1.146	0.364	0.310	
Cluster4 × other vegetables	0.014	-0.127	0.154	0.850		-0.021	-0.231	0.190	0.847		-0.213	-0.565	0.139	0.235	
Cluster1 × fruits	0.417	-0.055	0.889	0.083	*	-0.495	-1.204	0.214	0.171		0.038	-1.151	1.227	0.950	
Cluster2 × fruits	-0.289	-0.970	0.392	0.404		0.308	-0.715	1.330	0.555		-1.085	-2.796	0.626	0.214	
Cluster3 × fruits	-0.003	-0.479	0.473	0.990		-0.214	-0.928	0.500	0.557		-1.266	-2.458	-0.074	0.037	**
Cluster4 × fruits	-0.007	-0.197	0.183	0.945		0.256	-0.029	0.540	0.078	*	-0.134	-0.611	0.344	0.583	
Cluster1 × mushrooms	-0.293	-0.847	0.261	0.299		0.207	-0.625	1.040	0.625		0.253	-1.141	1.646	0.722	
Cluster2 × mushrooms	-0.229	-0.706	0.248	0.347		0.061	-0.656	0.777	0.868		1.554	0.359	2.749	0.011	**
Cluster3 × mushrooms	0.089	-0.511	0.690	0.771		-0.101	-1.002	0.801	0.826		-0.941	-2.449	0.567	0.221	
Cluster4 × mushrooms	-0.013	-0.151	0.124	0.848		-0.131	-0.337	0.075	0.211		0.226	-0.119	0.571	0.199	
Cluster1 × seaweeds	-0.257	-0.971	0.458	0.481		0.286	-0.787	1.358	0.601		0.063	-1.734	1.860	0.945	
Cluster2 × seaweeds	0.523	-0.531	1.576	0.330		-0.583	-2.165	0.999	0.470		1.142	-1.507	3.791	0.398	
Cluster3 × seaweeds	0.222	-0.103	0.548	0.180		0.315	-0.173	0.804	0.205		-0.646	-1.464	0.172	0.122	
Cluster4 × seaweeds	-0.018	-0.162	0.126	0.804		0.079	-0.137	0.295	0.472		-0.174	-0.536	0.187	0.344	
Cluster1 × seafood	0.287	-0.174	0.747	0.223		-0.023	-0.715	0.670	0.949		-0.346	-1.505	0.814	0.559	
Cluster2 × seafood	0.237	-0.256	0.730	0.345		0.521	-0.219	1.260	0.167		0.512	-0.728	1.752	0.418	
Cluster3 × seafood	0.103	-0.265	0.471	0.582		0.178	-0.375	0.731	0.527		-0.267	-1.192	0.659	0.571	
Cluster4 × seafood	0.099	-0.042	0.241	0.169		0.028	-0.185	0.241	0.797		0.212	-0.144	0.568	0.242	
Cluster1 × meat	0.382	-0.095	0.858	0.116		1.019	0.306	1.732	0.005	***	-0.161	-1.362	1.039	0.792	
Cluster2 × meat	-0.169	-0.427	0.090	0.201		-0.388	-0.775	-0.001	0.050	**	0.579	-0.069	1.228	0.080	*
Cluster3 × meat	0.216	-0.153	0.585	0.251		-0.058	-0.612	0.497	0.838		-0.772	-1.699	0.155	0.103	
Cluster4 × meat	0.133	-0.006	0.271	0.061	*	0.070	-0.139	0.279	0.510		-0.298	-0.647	0.052	0.095	*
Cluster1 × eggs	0.006	-0.492	0.504	0.982		1.075	0.331	1.818	0.005	***	0.721	-0.530	1.972	0.258	
Cluster2 × eggs	0.041	-0.299	0.382	0.811		0.254	-0.256	0.764	0.329		-0.522	-1.377	0.332	0.230	
Cluster3 × eggs	0.174	-0.161	0.510	0.309		-0.044	-0.548	0.460	0.865		-0.286	-1.130	0.558	0.506	
Cluster4 × eggs	0.084	-0.044	0.213	0.199		0.076	-0.117	0.270	0.441		0.040	-0.284	0.365	0.807	
Cluster1 × dairy products	0.064	-0.390	0.518	0.782		0.176	-0.506	0.857	0.613		-0.814	-1.954	0.327	0.162	
Cluster2 × dairy products	-0.062	-0.781	0.658	0.866		1.249	0.173	2.325	0.023	**	1.148	-0.659	2.954	0.213	
Cluster3 × dairy products	0.328	-0.012	0.668	0.059	*	-0.063	-0.575	0.449	0.810		-0.499	-1.355	0.358	0.253	
Cluster4 × dairy products	0.001	-0.141	0.143	0.986		-0.138	-0.351	0.075	0.204		-0.061	-0.418	0.297	0.738	
Cluster1 × alcohol	0.261	-0.088	0.611	0.143		-0.630	-1.154	-0.106	0.018	**	0.588	-0.291	1.468	0.190	
Cluster2 × alcohol	-0.263	-0.797	0.271	0.334		-0.224	-1.026	0.579	0.585		0.032	-1.312	1.376	0.962	
Cluster3 × alcohol	-0.116	-0.541	0.310	0.593		0.013	-0.626	0.652	0.967		-0.316	-1.386	0.754	0.562	
Cluster4 × alcohol	-0.010	-0.146	0.127	0.889		0.117	-0.088	0.322	0.262		-0.019	-0.363	0.324	0.913	
Female	-0.417	-0.875	0.041	0.074	*	-0.034	-0.723	0.655	0.923		1.957	0.812	3.102	0.001	***
Age	0.006	-0.004	0.016	0.258		-0.008	-0.023	0.008	0.317		0.009	-0.017	0.035	0.498	
Living alone	0.168	-0.124	0.461	0.259		0.486	0.047	0.925	0.030	**	0.143	-0.594	0.880	0.703	
Vigor	0.027	-0.024	0.078	0.298		-0.356	-0.427	-0.284	0.000	***	-0.117	-0.244	0.010	0.072	*
Irritability	0.187	0.128	0.245	0.000	***	0.159	0.070	0.248	0.000	***	0.023	-0.127	0.174	0.763	
Fatigue	0.171	0.111	0.232	0.000	***	0.231	0.139	0.322	0.000	***	0.510	0.360	0.661	0.000	***
Anxiety						0.618	0.519	0.718	0.000	***	0.235	0.052	0.417	0.012	**
Depression	0.274	0.230	0.319	0.000	***						0.265	0.144	0.385	0.000	***
Physical	0.037	0.008	0.066	0.012	**	0.094	0.051	0.137	0.000	***					
Const.	-0.484	-1.513	0.544	0.356		5.302	3.806	6.797	0.000	***	10.334	7.859	12.809	0.000	***

* p ≤ 0.1, ** p ≤ 0.05, *** p ≤ 0.005
